# Influence of a Short‐Term Attention Intervention on the Attentional Skills of Toddlers With Suspected or Confirmed Autism Spectrum Disorder

**DOI:** 10.1111/cdev.70033

**Published:** 2025-08-22

**Authors:** Lori‐Ann R. Sacrey, Lonnie Zwaigenbaum, Isabel M. Smith, Jessica A. Brian, Sam Wass, Emily J. H. Jones, Mark H. Johnson

**Affiliations:** ^1^ Autism Research Centre, Glenrose Rehabilitation Hospital and Department of Pediatrics University of Alberta Edmonton Alberta Canada; ^2^ IWK Health and Departments of Pediatrics and Psychology and Neuroscience Dalhousie University Halifax Nova Scotia Canada; ^3^ Bloorview Research Institute and Department of Pediatrics University of Toronto Toronto Ontario Canada; ^4^ Department of Psychological Sciences University of East London London UK; ^5^ Centre for Brain and Cognitive Development, Department of Psychology, Birkbeck University of London London UK; ^6^ Department of Psychology University of Cambridge Cambridge UK

**Keywords:** attention, autism, joint attention, toddlers, virtual

## Abstract

Examination of the effectiveness of an attention intervention using a randomized controlled trial for toddlers with suspected or confirmed autism spectrum disorder (ASD). Data was collected from Alberta, Ontario, and Nova Scotia, Canada between February 2018 and February 2020 (halted due to COVID‐19 pandemic). Participants were 35 toddlers randomized to the attention condition (age at start: 25.49 + 3.91 months; 29 boys; mother’s ethnicity: 65% white) and 34 toddlers randomized to a control condition. (age at start: 26.32 + 3.55 months; 24 boys; mother’s ethnicity: 29% white). The results suggest that the attentional skills can be improved by a computer‐based attention intervention, which in turn affects behavior observed in a real‐world setting.

Autism spectrum disorder (ASD) is a neurodevelopmental condition that is diagnosed in roughly 1 in 36 American children (Maenner et al. 2023) and is characterized by differences in social communication and the presence of restricted/repetitive interests and/or behaviors (American Psychiatric Association 2013). Treatment approaches for toddlers with ASD share many core elements, such as communication, social attention, imitation, affect sharing, and play, amongst others, but vary in emphasis on targets, intensity, duration, setting, and therapeutic method (therapists, caregivers, or a combination of both; Frost et al. 2020; Brian et al. 2016; Sandbank et al. 2020). Therapist‐led programs can yield clinical gains when intensely delivered over years (e.g., Dawson et al. 2010; Rogers et al. 2019), yet they tend to be costly and have long waitlists due to time involved and therapist capacity.

Caregiver‐led therapy for toddlers with ASD has a growing body of research support—these approaches involve a therapist providing education and coaching to caregivers who then engage in the intervention with their child (Nevill et al. 2018). These approaches leverage the caregiver‐child relationship as its foundation from which one builds upon skills that are guided by developmentally appropriate play‐based behaviors undergirded by the child's home environment (Brian et al. 2022). One such caregiver‐led intervention for toddlers with ASD, the Social ABCs, provides caregivers with education and strategies to promote child responsivity (attending to the caregiver) and expressive communication (responding to the caregiver). This 12‐week intervention has shown gains in measures of language and social development; yet additional benefits may be achievable by enhancing attention control, which is not targeted by the Social ABCs or other interventions for toddlers with ASD (Brian et al. 2015).

## 1 Attention as an Early Target

Attentional differences have been widely described in autistic individuals (Orekhova and Stroganova 2014) from as early as the first year of life. For example, Elison et al. (2013) and Bryson et al. (2018) demonstrated that infants under 12 months who were later diagnosed with ASD took longer to look away from one stimulus to engage attention on a newly appearing peripheral stimulus (visual disengagement) compared to toddlers without ASD. Further, Sacrey et al. (2023) identified differences in sustained attention (time spent looking at interesting and boring still images) and measures of joint attention (responding to or initiating bids with a partner; Mundy and Hogan 1996) in 2‐year‐old autistic toddlers compared to neurotypical peers.

Early learning environments often contain a complex mix of sights and sounds, and multiple social partners talking or acting at once. As a result, they can be overwhelming for young learners. Attentional control, our ability to choose to which of these sights and sounds we direct our attention and which we ignore is essential for avoiding overload (Rueda et al. 2023), and, through this, for driving learning (Karmiloff‐Smith 1998; Wass et al. 2012). A stimulus‐rich environment requires us to modulate different aspects of attention to promote learning, including selecting stimuli to which to attend, sustaining attention on the selected stimuli while ignoring potential distractors, disengaging attention from one stimulus to attend to another, and alternating attention between two or more social and non‐social environmental stimuli (Scerif 2010). As such, disruptions in any of these aspects of attention may affect learning across a range of domains (Johnson 2012; Karmiloff‐Smith 2009). Attentional performance has been linked to early language development (Kannass and Oakes 2008; Rose et al. 2009), academic learning (Razza et al. 2010; Steele et al. 2012; Welsh et al. 2010), and initiating and maintaining social interactions (Mundy et al. 2009). As such, interventions that target different aspects of attention may facilitate developmental progress across a range of domains.

## 2 Attention Interventions for Toddlers

Various behavioral interventions exist for targeting attention in children of school age and beyond (Ogundele and Ayyash 2023; Sibley et al. 2023), yet interventions for toddlers and very young preschoolers are limited. One approach is a computer‐based intervention that incorporates gaze‐contingent eye‐tracking; the child visually interacts with stimuli that respond and adapt depending on where and how long the child looks on the screen (Wass et al. 2011). This method has been found to be feasible and effective in very young typically developing infants (age 10–14 months) who received attention training over a 2‐week period (Wass et al. 2011). Improvements in different aspects of attention for 12‐month‐old infants from a low socioeconomic background (Ballieux et al. 2016) or who were very preterm (Perra et al. 2022) were found following 5 weeks of attention training. Applying the same training with typical 9‐month‐olds led to transfer effects on a structured observation task assessing the infants' likelihood to respond to an adult's social‐communication cues (Forssman and Wass 2018). However, a recent study with infant siblings of children with ADHD showed no significant changes in attention following attention training (Goodwin et al. 2021). A feasibility study of attention training using this paradigm (Wass et al. 2011) with autistic children (average age 6 years) showed improvements in tasks of sustained attention, although some stimuli were posited to be less engaging or some tasks too easy for this age group (Powell et al. 2016). As such, we are confident that attention training in younger children (under 3 years of age) with suspected or confirmed ASD will show improvements following attention training.

## 3 Current Study

The purpose of the present study was to assess the performance of a brief attention training intervention in toddlers who were showing early features of ASD. Toddlers under 3 years of age were randomly assigned to either an intervention that targeted attention and working memory or a control condition that involved passive viewing of children's animated videos. Prior to and following the intervention, toddlers participated in gaze‐contingent computer‐based attention activities that measure sustained attention (maintaining gaze on a stimulus), disengaging attention (shifting attention from one visual stimulus to another), and cognitive control (inhibiting a previously learned rule to learn a new rule; per Goodwin et al. 2021). Activities were designed to be toddler‐friendly, using bright colors and animations to keep toddlers engaged. Toddlers also participated in an interactive (non‐computerized) joint attention task with an examiner before and after the attention training (Mundy and Hogan 1996). We hypothesized that toddlers in the attention training group would show (1) differences on the computer‐based tasks (e.g., increased looking durations to “interesting” stimuli during sustained attention and faster disengagement on attention tasks, as well as greater saccade accuracy on a cognitive control task) and (2) transfer effects on an interactive joint attention task (e.g., increased frequencies of initiating and responding to joint attention) compared to toddlers who received the control condition.

## 4 Methods

### 4.1 Participants

Participants were toddlers between 12 and 30 months of age who displayed clinically significant concerns related to ASD, which could not be accounted for by a specified neurological or genetic condition. Clinically significant concern was determined from scores on the eligibility assessment (described below), parents' reported concerns, and clinical judgment. All participants were born between 37‐ and 42‐weeks' gestation, with birth weights greater than 2500 g, and no reports of birth complications. Participants were recruited through major Canadian diagnostic and treatment centers in Alberta, Ontario, and Nova Scotia. Recruitment materials were shared with community pediatricians, early intervention services, and on social media. Interested caregivers were screened for eligibility characteristics including: (i) child is between 12 and 30 months of age; (ii) child had a birthweight of at least 2500 g and was born at 36 weeks or more; (iii) child does not have known neurological or genetic disorders; (iv) child is not involved in any other services, interventions, or therapies; (v) parent did not have prior training in Pivotal Response Training (PRT) or Social ABC's; and (vi) parent reported concerns of social communication challenges. Families who met eligibility criteria were invited for a clinical assessment to measure ASD‐related features. Participants underwent an assessment using standard clinical assessment tools/processes overseen by a qualified clinician to explore eligibility using the Autism Diagnostic Observation Schedule—2nd Edition (Lord et al. 2012), the Mullen Scales of Early Learning (Mullen 1995), and the Parent‐Rated Observation of Communication, Emotion, and Social Skills (PROCESS; Bryson et al. 2006) to confirm ASD‐related concerns. Families were invited to participate if they met standardized cut‐off scores on the ADOS‐2 Toddler module for “mild‐to‐moderate concern” and/or clinical judgment following observations during the ADOS‐2 and Mullen that identified social communication challenges. The research ethics boards at the University of Alberta, Holland Bloorview Research Institute, and IWK Health Centre approved this study, and all families gave written informed consent prior to enrollment.

### 4.2 Demographics

As displayed in Table 1, the two groups did not differ on sex (*χ* ^2^ = 2.71, *p* = 0.10), birth order (*χ* ^2^ = 3.12, *p* = 0.25), having an older sibling with ASD (*χ* ^2^ = 3.24, *p* = 0.07), or father's ethnicity (*χ* ^2^ = 5.51, *p =* 0.06). Groups differed on mothers' ethnicity (*χ* ^2^ = 9.06, *p* = 0.01); as such, mother's ethnicity was included as a potential covariate.

**TABLE 1:** Demographic and clinical characteristics.

	Control	Attention training	Statistics
*n*	35	34	
Age	26.32 (±3.55) months	25.49 (±3.91) months	*z =* −0.67, *p* = 0.50
Sex	24 boys: 11 girls	29 boys: 5 girls	*χ* ^2^ = 2.71, *p* = 0.10
Birth order	Range: 1–5	Range 1–5	*χ* ^2^ = 3.12, *p* = 0.25
Older sib with ASD	Yes—6% No—83% No response—11%	Yes—21% No—70% No response—9%	*χ* ^2^ = 3.24, *p* = 0.07
Mother's ethnicity	White—29% BIPOC—54% Not given—17%	White—65% BIPOC—26% Not given—9%	***χ*** ^**2**^ **= 9.06, *p* = 0.01**
Father's ethnicity	White—34% BIPOC—46% Not given—20%	White—58% BIPOC—21% No given—21%	*χ* ^2^ = 5.51, *p* = 0.06
Mullen Scales of Early Learning—age‐equivalent scores			
Visual reception (*n*) (*M* ± SD)	33 16.64 (5.99)	33 18.91 (7.17)	*z* = −0.93, *p* = 0.35
Fine motor (*n*) (*M* ± SD)	19 16.26 (4.64)	24 18.21 (5.70)	*z* = −1.39, *p* = 0.17
Receptive language (*n*) (*M* ± SD)	21 9.00 (5.53)	23 13.61 (8.58)	***z* = −2.24, *p* = 0.03**
Expressive language (*n*) (*M* ± SD)	34 12.44 (6.63)	32 15.13 (6.99)	***z* = −1.99, *p* = 0.04**
Autism Diagnostic Observation Schedule—toddler module—severity scores			
*n*	32	33	
Social affect (*M* ± SD)	15.88 (5.10)	14.33 (4.81)	*z* = −1.62, *p* = 0.11
RR behavior (*M* ± SD)	4.88 (1.90)	4.36 (2.00)	*z* = −0.94, *p* = 0.35
Total (*M* ± SD)	20.75 (6.15)	18.70 (5.90)	*z* = −1.79, *p* = 0.07
Parent Rated Observation of Communication, Emotion, and Social Skills			
*n*	31	31	
Total score (*M* ± SD)	23.97 (9.14)	22.00 (9.38)	*z* = −1.30, *p* = 0.19

Abbreviations: BIPOC = Black, Indigenous, and People of Color; M = mean; SD = standard deviation. Values provided in bold indicate a statistically significant result.

#### 4.2.1 Clinical Assessment

At the baseline assessment, no group differences were obtained for ADOS‐2 Total (*p* = 0.07), Social Affect (*p* = 0.11), or RRB (*p* = 0.35) severity scores. On the Mullen, groups did not differ on Visual Reception (*p* = 0.36) or Fine Motor age equivalents (*p* = 0.17), but Receptive (RL; *p* = 0.03) and Expressive (EL; *p* = 0.04) Language age equivalents were higher in the attention group compared to the control group. The PROCESS parent questionnaire total scores did not differ between groups (*p* = 0.19).

### 4.3 Trial Design

Following informed consent and the clinical assessment, participants were randomly allocated in a 1:1 ratio to an attention training (i.e., intervention) or control condition (i.e., placebo). A random allocation sequence was created in blocks of four (two attention, two control) using randomly generated numbers from the uniform distribution. These were generated using Microsoft Excel and constrained the attention/control ratio to 1:1. The examiner responsible for delivering the attention training or control condition received the allocation in a sealed opaque envelope when a new participant was due to begin testing (i.e., after caregivers provided informed consent). Families completed two sessions of either attention training or control condition per week over a 4‐week period, for a total of eight training/control sessions. Prior to and following the intervention, participants completed an *Attention Assessment* addressing four aspects of attention: selecting stimuli to attend to, sustaining attention toward stimuli in the environment, disengaging from one stimulus to attend to a nearby stimulus, and alternating attention between two or more stimuli.

As part of this study, all families were invited to participate in a parent‐mediated intervention, the Social ABCs (Brian et al. 2016), following completion of the post *Attention Assessment*. The overarching goal of this RCT was to determine whether participation in an attention intervention primed benefits of participation in the Social ABCs (data not included in this paper). The current paper shares the data from the attention training portion of the RCT to understand the short‐term effects of attention training on various measures of attention. Data collection for the *Attention Assessment* and subsequent intervention began in October 2017 and ended in March 2020 (due to COVID‐19 pandemic).

### 4.4 Sample Size Calculation

Sample size was calculated based on a conservative estimate of effect sizes (0.5 for each primary outcome: disengage latencies and joint attention), as this study involved a novel application of the Attention Assessment (described below). Wass et al. (2011) reported effect sizes of 0.68 for disengage latency and 0.54 for joint attention in his previous report using the Attention Assessment. Calculation results indicated 63 participants per group, plus oversampling of 20% to account for attrition, for 150 participants (75 per group).

**FIGURE 1:**
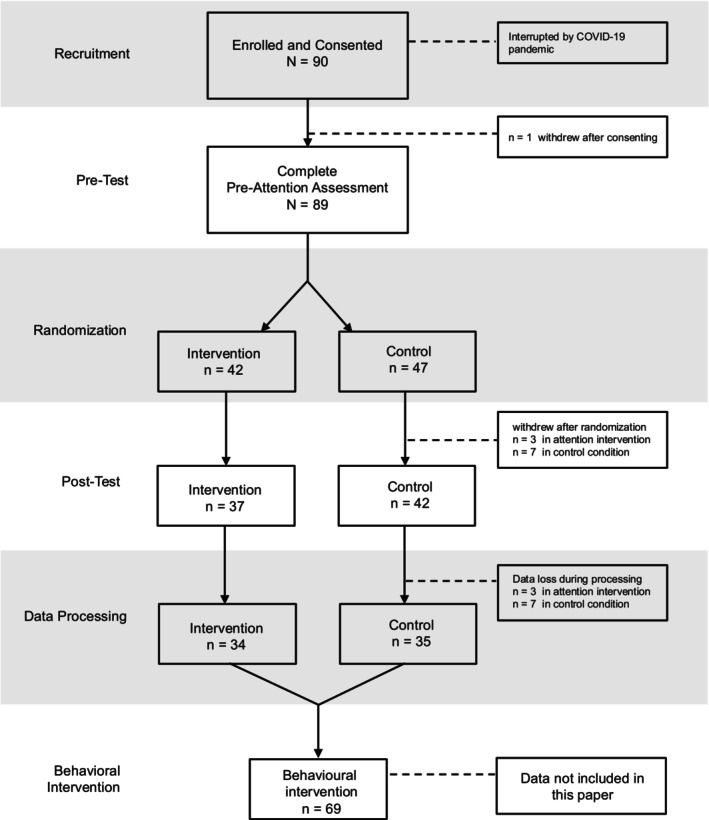
CONSORT diagram of participant flow.

The CONSORT diagram of participant flow is shown in Figure 1. Data collection was to take place over 3 years but was interrupted during year 2 by the COVID‐19 pandemic, halting recruitment. In total, 90 children were recruited, with 79 completing the pre‐ and post‐Attention Assessment and 11 withdrawing before completing the attention intervention/control condition and post‐Attention Assessment. Data loss due to incomplete gaze‐contingent data and movement errors resulted in data from a total sample of 69 children included in the following analyses. Post hoc power calculations (i) ranged from 0.13 (mean look to boring trials) to 0.56 (mean look interesting trials) on the Sustained Attention Task; (ii) ranged from 0.14 to 0.39, with 0.24 for the overlap trials on the Disengage Task; (iii) ranged from 0.07 to 0.72, with 0.72 for the number of correct anticipatory saccades on the Cognitive Control task; and (iv) ranged from 0.05 to 0.72, with 0.72 for pointing and responding to the book on the Joint Attention task of the post‐Attention Assessment.

### 4.5 Clinical Assessment Measures

The Mullen Scales of Early Learning (Mullen), Autism Diagnostic Observation Schedule—2nd Edition (ADOS‐2), and Parent‐Rated Observation of Communication, Emotion, and Social Skills (PROCESS) were completed to screen for features of ASD. The Mullen (Mullen 1995) is a measure for children aged 0–60 months that assesses Visual Reception, Receptive Language, Expressive Language, Fine Motor, and Gross Motor abilities, yielding an Early Learning Composite (ELC) comprising the first four scales. We administered all but the Gross Motor domain for this study. The ADOS‐2 (Lord et al. 2012) was administered by a research‐reliable examiner. The ADOS‐2 includes standardized activities and “presses” intended to elicit communication, social interaction, imaginative use of play materials, and repetitive behavior. The Toddler module was administered and Social Affect (SA), Restricted and Repetitive Behavior (RRB), and Total algorithm scores were derived. The PROCESS (formerly the Autism Parent Scale for Infants [APSI]; Bryson et al. 2006) is a 26‐item forced‐choice (“yes,” “sometimes,” “no”) parent‐report questionnaire that covers a range of behavioral features of ASD in infants aged 6–24 months. More responses indicating the presence of ASD‐like behavior result in a higher score. The PROCESS has fair to excellent internal consistency (range: 0.77 at 6 months to 0.92 at 24 months), as reported in a sample of children at increased likelihood of ASD (i.e., younger siblings of autistic children), who did or did not receive a diagnosis at 36 months (Sacrey et al. 2018). These measures were all completed prior to the participants' exposure to the computer‐based tasks.

### 4.6 Attention Assessment

The *Attention Assessment* comprised three computerized tasks and one interactive task. It was administered to participants twice: prior to randomization and following completion of the intervention.

#### 4.6.1 Computer‐Based Tasks

The computer‐based attention tasks use gaze‐contingent animations that rely on tracking of eye movements. Toddlers viewed the Disengagement, Sustained Attention, and Cognitive Control tasks on a computer screen. Data were collected using a Tobii Pro X3‐120 Eye Tracker. During the computer‐based tasks, toddlers sat in a high chair or on their parents' laps, with parents wearing infrared‐blocking glasses to ensure only the toddlers' eyes were tracked. The attention tasks were presented on a Dell 19‐in. monitor with a screen resolution of 1024 × 768. The tasks were run using MATLAB scripts and previously tested with neurotypical infants (Goodwin et al. 2016). Prior to testing, toddlers completed a calibration sequence to ensure their eyes were being correctly tracked. Children were seated inside a large white canvas tent with their caregiver across from a computer monitor mounted on a stand. The tent served to minimize distractors as the computer monitor was the only salient stimuli inside the tent (except for the caregiver). The examiner sat outside the tent to run the computer program. Children (and their eye movements) were monitored using a webcam mounted to the top of the computer monitor. No verbal instructions were given to the toddlers to attend to the screen.

Data were pre‐processed by removing any offscreen gaze and setting all missing gaze samples to NaN (null). The robustness of eye‐tracking was quantified by calculating the duration (in seconds) of usable fragments of eye‐tracking data obtained during recordings. Eye‐tracking data tend to “flicker” on and off during recording, as is reported in detail elsewhere (Leppänen et al. 2015; Wass et al. 2014), due to certain elements of the information required to calculate the infant's position of gaze (pupil, corneal reflection, and the position of the head in 3D space) being unavailable or insufficiently robust by the image processing algorithms built into the eye‐tracker, resulting in NaN values being returned.

Initial processing of the data were conducted using the following method: if data from two eyes were present, we averaged the gaze coordinates; if data from only one eye were present, we used those. Areas of interest (AOI) were identified only on the x‐axis. We obtained AOI metrics (looking time, first entry time) for left and right AOIs. We calculated reaction times to AOIs and identified whether anticipatory saccades occurred before the stimuli appeared (erroneous vs. valid trials). We calculated whether anticipatory saccades were made to the correct or incorrect stimuli and calculated the accuracy and precision to the fixation stimulus. Processing steps taken for each task following pre‐processing are described in their respective sections.

##### 4.6.1.1 Sustained Attention

Four still images were presented in four blocks interspersed with disengagement task blocks (similar to Goodwin et al. 2021; see below). The first block contained a “boring” image (low‐detail, monochrome outlines of a diamond and a cross); blocks two and three contained an “interesting” image (attractive, detailed images of flowers and fish), and the fourth block was a boring image. Trials started once the participant fixated on a central target, automatically triggering the picture to appear. Trials ended automatically when the participant looked away from the screen for 1 s or more. Following the end of a trial, a fixation target and brief (< 1 s) auditory stimulus were presented. If the participant fixated on the target, the next trial started immediately; if not, a sequence of different fixation targets and auditory cues was repeated. Each block consisted of the same picture presented five times, for a maximum of 120 s per trial. Background instrumental music played during this task that did not vary in sync with the visual displays. Four duration variables (all in seconds) for Sustained Attention were extracted:

- Peak look—longest look
- Minimum look—shortest look
- Mean look—average look
- Looking range—peak look minus the minimum look

##### 4.6.1.2 Disengagement Task

Four blocks of 12 trials each were interspersed with the sustained attention task (see above). All stimuli were 3 cm × 3 cm (2.86° × 2.86° at 60 cm viewing distance). Each trial started with the onset of a central stimulus (CS); a cartoon image of a spinning ball accompanied by an alerting sound. This pulsed on screen at 3 Hz between 3 and 5 cm (2.86°–4.77°) until fixated by the participant. The CS then rotated at 500° per second for a random 500–700 ms inter‐stimulus interval (ISI). After the ISI, a 200 ms baseline, gap, or overlap period began, during which the stimuli varied according to condition. In the Baseline and Overlap conditions, the CS remained on screen, and in the Gap condition, the CS disappeared from the screen. After this 200‐ms period, the peripheral stimulus (PS) was presented. In the Baseline condition, the CS disappeared from the screen at the same time as the PS appeared. In the Overlap condition, the CS continued to be presented for the rest of the trial. The PS was a cartoon cloud (accompanied by a sound) that appeared on either the left or the right side of the screen, 3 cm (2.86°) from the edge, rotating at 500° per second until fixated by the participant. A “reward” stimulus was then presented at the location of the PS for 1000 ms. The reward was a randomly chosen colorful cartoon image of a star, a sun, a dog, a cat, a pig, a tiger, or a tortoise, accompanied by a playful sound. The reward stimulus was animated to spin on the spot, spin and shrink, or pulse.

Data were analyzed offline. Each trial was inspected automatically to determine trial validity and calculate a saccadic reaction time (SRT) to shift attention from the CS to the PS, relative to PS onset. A trial was valid if the following conditions were met: (1) child gaze fell on the CS; (2) no gaps of missing data longer than 200 ms were present during the CS period (before PS onset); (3) at least one sample of gaze was on the CS within 50 ms of PS onset; (4) no gaps of missing data longer than 100 ms were present during the PS period (between PS onset and reward onset); (5) SRT was longer than 150 ms and shorter than 1200 ms; (6) gaze did not go to the opposite side of the PS; (7) gaze did not enter the PS area of interest after engagement with the CS but before PS onset (per Goodwin et al. 2021). Trials that failed any of these criteria were invalidated and removed from further analysis. Mean SRTs were calculated for each condition separately, using only average reaction time in ms for valid baseline, gap, or overlap trials.

##### 4.6.1.3 Cognitive Control

Participants were presented with two blocks of 18 trials. Trials began after the participant fixated a central gaze‐contingent stimulus. Two blank rectangles (17 cm × 12.5 cm, 16.1° × 11.9° at 60 cm) were presented 0.5 cm (0.48°) from each edge of the screen and vertically centered. These remained on screen until either one of the rectangles was fixated by the participant or 2000 ms elapsed. At this point, one rectangle was replaced by video of the same dimensions, a 2 s clip of the animated children's TV program Peppa Pig. After the 2 s clip played, the screen was blanked and the next trial began with another gaze‐contingent fixation stimulus (based on Wass et al. 2011).

The video was displayed on the same side of the screen as the participant's gaze in the first trial. For subsequent trials, the participant could make an anticipatory saccade to the blank rectangle on this side, at which point the video would continue playing. The presence and speed of this anticipatory saccade were the main dependent variable. The second trial of each block marked the beginning of the “learning” phase. The side on which the video clip played remained fixed through this phase, which ended after a total of nine trials. After this, the “reversal” phase began, in which the correct side—the side on which the video would play—was reversed. On the first trial of the reversal phase, the participant was not aware that the reversal had occurred until the video played. All subsequent trials in the reversal phase proceeded as described above, except for the reversed side. We continued to record the presence and speed of an anticipatory saccade to the correct (newly reversed) side. The reversal phase also lasted for nine trials. To continue to the reversal phase, participants had to make at least three sequential saccades to the correct side out of a maximum of nine trials.

Data were analyzed offline. Raw continuous gaze‐contingent data were segmented into epochs representing each trial for each participant. The first trial of each phase, in which the participant was not yet aware of the correct side, was discarded. For all other trials, any looks that were away from the computer screen were marked as missing; left‐ and right‐eye data were averaged for samples in which binocular data were available; otherwise, we used monocular (detected eye) data. For each participant and for each phase, we calculated the number of looks to the correct and incorrect sides during the learning and reversal phases, as well as the proportion of correct anticipatory saccades to the correct side during the learning and reversal phases.

#### 4.6.2 Interactive Task

The *Early Social Communication Scales* (*ESCS*; Mundy and Hogan 1996) is a 20‐min structured interaction between the child and examiner that is used to assess skills that typically develop between the ages of 8 and 30 months. The ESCS targets three domains: social interaction (e.g., appeals for toy, turn‐taking), joint attention (e.g., pointing, alternating gaze), and behavioral regulation (i.e., child's behavior when a toy ceases to function or is placed out of reach). The ESCS has reliably detected functional differences in children with autism and developmental delays (Mundy et al. 1990). A video‐recorded abridged version (Sacrey et al. 2023) of the ESCS (hereafter referred to as the mini‐ESCS or M‐ESCS) was used in this study to measure joint attention (e.g., points, gazes). Specifically, we coded:

1. During toy play and toy spectacle (18 trials):
  - *Low‐level initiating joint attention* (*IJA*)—(i) child makes *eye contact* with the examiner while manipulating or touching an inactive toy; and (ii) child *alternates eye contact* between the active toy and examiner
  - *High‐level IJA—*child points at *active toy* with or without eye contact
2. Book (six trials) and Poster tasks (eight trials):
  - *Responding to joint attention* (*RJA*)—child's gaze turns to picture in book or to near (head turn to side) or far (look behind shoulder) posters following examiner's point
  - *Pointing*—child points to pictures in the book or near or far posters *before* the examiner has pointed; pointing occurs with or without eye contact.
  - *Point in imitation—*child produces a point within 5 s of an examiner's point
3. Bids to caregiver: any unprompted joint attention behavior directed to caregiver (e.g., eye contact, alternating eye contact, pointing with or without eye contact).

Twenty percent of all M‐ESCS videos were double‐coded for the calculation of inter‐rater reliability using intraclass correlations (ICC) with a two‐way mixed model evaluating absolute agreement on item‐level scores. Cicchetti's (1994) guidelines classify ICC scores of less than 0.40 as “poor,” between 0.40 and 0.59 as “fair,” between 0.60 and 0.74 as “good,” and between 0.75 and 1.00 as “excellent.” ICCs were excellent for each measure, at 0.98 for low‐level IJA, 0.93 for high‐level IJA, 0.99 for pointing during books and posters, and 0.99 for bids to caregiver.

### 4.7 Intervention

The tasks that comprised the attention intervention were those used by Wass et al. (2011) and Goodwin et al. (2021). These tasks respond adaptively to the child's performance, such that the tasks become harder as the child correctly completes each level of difficulty within the “game.” If the child's performance begins to decrease, the game adapts the difficulty level to ensure the child continues to experience success and attend to the “game.” This contrasts with the tasks in the Attention Assessment, such as the gap‐overlap task, which do not change in difficulty depending on the child's performance. The intervention targeted three areas of attention: goal maintenance, working memory, and target searching. For a complete description of each “game” within the attention intervention, see the Supporting Information S1.

#### 4.7.1 Goal Maintenance

An animated character (smiling pink character or butterfly) appeared on screen and began to fly following visual fixation. Moving distractors (clouds, trees, etc.) appeared around the character in the child's peripheral visual field; if the child looked away from the character to any of these moving (and therefore salient) distractors, the character stopped flying and the distractors disappeared. Once the child re‐fixated on the character, it would fly again, and the cycle repeated. Performance was calculated based on the child's fixation on the character weighted by the difficulty level (number and salience of distractors present).

#### 4.7.2 Working Memory

An animated character moved inside one of two (or more) squares on the screen and then all squares were covered to hide the location of the character. The screen then turned black, and a central stimulus appeared (a ball moving from top to bottom) to attract the child's attention. The covered squares reappeared. If the child looked to the square where the character had initially appeared, then a reward sequence was initiated, and the character was revealed. The “found” character remained on screen and a new character appeared and moved into the empty square. The screen then became black and the central stimulus (moving ball) reappeared. The two squares then reappeared with the first “found” character still visible inside the square and the square where the second character had appeared was occluded. When the child looked at the blank occluded square, a reward sequence was initiated, and the second character was revealed. Performance was calculated by calculating the reciprocal of the response time to look at the correct target. Thus, children who were faster in meeting task demands obtained higher scores.

#### 4.7.3 Target Searching

One of five animated characters was presented on screen with eight smaller distractors (stars, planets, clouds). If the child looked to the character within 3000 ms, they received an animation as a reward. The target character changed from trial to trial. The salience of the distractors changed adaptively; at lower difficulty, the distractors were smaller, static, and identical to each other and dissimilar from the targets. At higher difficulty, they were varied, moving, brightly colored, thus similar to the target character. Performance was calculated using the reciprocal of children's response time to find the correct target weighted by the difficulty level. In this way, infants obtained higher scores for succeeding in the task faster when the task presented higher demands.

### 4.8 Control Condition

The control condition consisted of 2–3‐min clips from age‐appropriate children's television programs, including Peppa Pig, Ben and Holly, Pingu, Postman Pat, In the Night Garden, and Boj. When participants' attention to the screen waned, examiners switched the clip to re‐attract the toddler's attention to the screen. The control videos were presented on the same equipment as the attention training, with a key difference that the control videos did not change contingent on the toddler's eye movements.

### 4.9 Statistical Analyses

Groups (attention, control) were compared on demographic characteristics and clinical measures to identify potential covariates using chi‐square for categorical data and Mann–Whitney *U* analyses for continuous data (*p*'s < 0.05). Because a substantial proportion of children did not meet raw score minimum cut‐offs to convert to *T*‐scores on the Mullen language subscales (25% of the sample on both Receptive and Expressive Language), we used age‐equivalents to maximize usable data. To identify covariates for the analyses, Spearman correlations were run on significant demographic variables and clinical measures to examine their relations to each outcome variable in the post‐*Attention Assessment* (*p*'s < 0.05).

Groups were compared on the post‐*Attention Assessment* using a series of mixed models to maximize data usability. For each model, the pre‐*Attention Assessment* variable was included as a covariate. Demographic characteristics or clinical measures were also included as covariates in mixed model analyses when a significant association with the post*‐Attention Assessment* variable (e.g., caregiver ethnicity) was found. See Table S2 for variables included in each model.

Within the group who received the intervention, facilitators of average change in attention training performance over the eight sessions were explored using Spearman rho correlations, including amount of training attended (number of sessions attended, average training time), as well as baseline scores on the Mullen, ADOS‐2, and PROCESS. The average change in attention training performance was calculated by transforming the performance measures for each task into *z*‐scores. Performance was assessed by (i) averaging the training games together to calculate a composite for each participant who received the intervention, as well as (ii) calculating a composite rate of change across all sessions and all games played. We also compared the performance on each game individually to determine whether certain games contributed more to change scores. To control for the number of comparisons, only correlations with *p* < 0.01 were considered significant.

## 5 Results

### 5.1 Group Differences on the Baseline Assessment

#### 5.1.1 Exploration of Covariates to Include in Statistical Comparisons

Pearson's correlations were used to assess the relationship between Mullen age‐equivalent scores and M‐ESCS joint attention measures as joint attention and language are conceptually related (see the meta‐analysis by Bottema‐Beutel 2016). As shown in Table S1, there were significant associations between Mullen age‐equivalent scores and measures on the M‐ESCS (eye contact, pointing, pointing in imitation, responding to the book task, and responding to near posters). As such, we did not include Mullen as a potential covariate. We used Spearman Rho correlations to assess the relation between mother's ethnicity and the post‐*Attention Assessment* variables. As shown in Table S2, mother's ethnicity was included as a covariate for alternate eye contact and the book task (Joint Attention). No other associations were significant.

### 5.2 Group Differences on Intervention Attendance

#### 5.2.1 Number of Sessions Attended

Groups did not differ in the number of sessions attended (*χ* ^2^ = 2.66, *p* = 0.62), with the attention group attending 6.88 (±1.18) training sessions and the control group attending 6.83 (±1.04) placebo sessions.

#### 5.2.2 Data Completion

There were no group differences for pre‐ and post‐*Attention Assessment* data availability (described below) for Sustained Attention (*p* = 0.69), Disengagement Task (*p* = 0.99), Cognitive Control (*p* = 0.28), or the Joint Attention (*p* = 0.64) tasks. The usable data for each task by group are provided in Table 2.

**TABLE 2:** Data availability by task and group.

Task	Control group	Attention group
	Pre *n*/post *n*	Pre *n*/post *n*
Sustaining attention		
Boring	36/34	32/32
Interesting	35/35	30/31
Disengagement task		
Baseline	19/18	18/18
Gap	19/18	18/18
Overlap	18/18	17/18
Cognitive control		
Learning	32/35	28/29
Reversal	32/33	28/29
Joint attention		
Initiating JA	32/30	28/28
Responding JA	29/29	28/27

### 5.3 Post‐Attention Assessment

#### 5.3.1 Computer Tasks

##### 5.3.1.1 Cognitive Control

During learning, the number of anticipatory saccades (regardless of if they were correct or incorrect) was not different between groups (*F* (1, 56) = 2.08, *p* = 0.16, *d* = 0.39); yet the number of *correct* anticipatory saccades (*F* (1, 56) = 5.07, *p* = 0.03, *d* = 0.60) differed between groups, with the attention group making more correct anticipatory saccades (5.67 ± 2.05) than the control group (4.51 ± 2.02). The proportion of correct anticipatory saccades (*F* (1, 56) = 4.70, *p* = 0.03, *d* = 0.58) differed between groups, with the attention group having a higher proportion of correct anticipatory saccades (0.76 ± 0.22) than the control group (0.65 ± 0.23).

During reversal, the number of anticipatory saccades (regardless of whether they were correct or incorrect) was not different between groups (*F* (1, 54) = 3.83, *p* = 0.06, *d* = 0.53); nor was the number of correct anticipatory saccades (*F* (1, 54) = 1.09, *p* = 0.30, *d* = 0.28) or the proportion of correct anticipatory saccades (*F* (1, 54) = 0.04, *p* = 0.85, *d* = 0.05). See Figure S1 for a visualization of the data.

##### 5.3.1.2 Sustained Attention

During boring trials, there was no group difference for peak look (*F* (1, 63) =0.46, *p* = 0.50, *d* = 0.18), mean look (*F* (1, 63) =0.25, *p* = 0.62, *d* = 0.13), minimum look (*F* (1, 63) =0.75, *p* = 0.39, *d* = 0.22), nor looking range (*F* (1, 63) =0.75, *p* = 0.39, *d* = 0.22).

During interesting trials, there was no difference for peak look (*F* (1, 60) = 2.85, *p* = 0.10, *d* = 0.44), mean look (*F* (1, 60) = 3.12, *p* = 0.08, *d* = 0.46), minimum look (*F* (1, 60) =0.44, *p* = 0.51, *d* = 0.17), nor looking range (*F* (1, 60) = 2.68, *p* = 0.11, *d* = 0.42). See Figure S2 for a visualization of the data.

##### 5.3.1.3 Disengagement Task

There was no group difference in saccadic reaction time for baseline trials (*F* (1, 31) = 1.32, *p* = 0.26, *d* = 0.41), gap trials (*F* (1, 31) = 0.03, *p* = 0.86, *d* = 0.20), overlap trials (*F* (1, 29) = 0.88, *p* = 0.36, *d* = 0.34), facilitation (*F* (1, 31) = 1.83, *p* = 0.19, *d* = 0.49), nor disengagement (*F* (1, 31) = 0.51, *p* = 0.48, *d* = 0.26). See Figure S3 for a visualization of the data.

#### 5.3.2 Behavioral Task

##### 5.3.2.1 Joint Attention

For initiating bids for joint attention (IJA), the number for eye contact (*F* (1, 52) = 3.35, *p* = 0.07, *d* = 0.51), alternative (*F* (1, 52) = 1.15, *p* = 0.29, *d* = 0.30), and total low‐level IJA events (*F* (1, 52) = 3.07, *p* = 0.09, *d* = 0.49) were not different between groups. The number of pointing events (*F* (1, 52) = 5.11; *p* = 0.03, *d* = 0.63) differed between groups, with the attention group producing more points (4.32 ± 7.17) than the control group (1.32 ± 2.71). The number of points with eye contact events (*F* (1, 52) = 0.54, *p* = 0.47, *d* = 0.20), showing to parents (*F* (1, 52) = 0.42, *p* = 0.52, *d* = 0.18), bid for caregiver attention (*F* (1, 52) = 0.08, *p* = 0.78, *d* = 0.25), and total high‐level IJA events (*F* (1, 52) = 0.001, *p* = 0.98, *d* = 0.01) were not different between groups.

For responding to joint attention, responding to examiners' points in a book (*F* (1, 48) = 5.20, *p* = 0.03, *d* = 0.63) differed between groups, with the attention group responding to more points (60.21% ± 34.67%) than the control group (35.00% ± 31.64%); pointing in imitation (*F* (1, 53) = 5.01, *p* = 0.03, *d* = 0.62) differed between groups, with the attention group producing more imitative points (1.79 ± 2.38) than the control group (0.61 ± 1.38). Responding to points at near posters (*F* (1, 54) = 4.17, *p* = 0.05, *d* = 0.57) was significantly different between groups, with the attention group responding to more points (42.50% ± 38.37%) than the control group (28.48% ± 27.95%); however, responding to far posters (*F* (1, 54) = 0.16, *p* = 0.70, *d* = 0.11) was not different between groups. See Figure S4 for a visualization of the data.

#### 5.3.3 Summary

On the computer‐based tasks, the attention group (i) had a higher number of correct anticipatory saccades and a higher percentage of correct anticipatory saccades to the target on the learning phase of the *Cognitive Control* task compared to the control group; (ii) were not different from the control group on *Sustained Attention*; and (iii) were not different from the control group on the *Disengagement Task*. On the behavioral task, the attention group (i) had a higher number of pointing events for initiating joint attention compared to the control group; and (ii) had a higher percentage of responding to examiner points in the book, points to near posters, and points in imitation for responding to bids for joint attention compared to the control group.

### 5.4 Exploration of Contributors of Attention Training Performance

Within the group who received the intervention, facilitators of average change in attention training performance over the eight sessions were explored.

#### 5.4.1 Training Performance

##### 5.4.1.1 Composite Change Scores

With respect to parameters of the training games, composite average change was significantly related to the number of training sessions attended (rho = 0.36, *p* = 0.009), with greater composite change associated with more sessions attended. There was no relationship between composite average change, composite rate of change, and total training time (*p*'s > 0.05). With respect to clinical characteristics, composite average change was significantly related to Mullen VR age‐equivalents (rho = 0.36, *p* = 0.009), with greater change associated with higher VR age‐equivalents at baseline.

##### 5.4.1.2 Individual Game Change Scores

Average change for each individual game was not associated with the number of training sessions attended (all *p*'s > 0.05) nor total training time (all *p*'s > 0.05). Mullen VR age‐equivalent was associated with average change for Stars (rho = 0.41, *p* = 0.01) and Mullen EL age‐equivalent was associated with average change for Butterfly (rho = −0.58, *p* < 0.001). Higher scores on ADOS‐2 Total severity (rho = −0.55, *p* = 0.003) and SA severity (rho = −0.41, *p* = 0.01) were associated with lower scores on Stars. Higher ADOS‐2 RRB severity scores were associated with lower scores on Windows (rho = −0.48, *p* = 0.003) and Stars (rho = −0.43, *p* = 0.008).

##### 5.4.1.3 Attention Assessment

Potential facilitators of change for the significant variables described above were explored by correlating change scores (i.e., *Attention Assessment* post score—pre score = change score) with features of the training environment, including total training time, average training per game, number of games completed, and number of sessions completed. We chose a composite score for these analyses to control for pre‐*Attention Assessment* performance, which may have differed between groups and would not have been impacted by intervention participation. Demographic characteristics, including baseline Mullen age‐equivalents, ADOS‐2 Total severity score, and total PROCESS score, were explored.

###### 5.4.1.3.1 Cognitive Control Task

The proportion of correct anticipatory saccades was associated with the composite rate of change (rho = −0.47 *p* = 0.001), suggesting that toddlers with a lower rate of change had smaller discrepancy scores (i.e., smaller differences in pre‐ and post‐proportions), which may have resulted from a ceiling effect. Numbers of correct anticipatory saccades (rho = 0.50, *p* = 0.006) and proportion of correct anticipatory saccades (rho = 0.57 *p* = 0.002) were associated with total PROCESS scores, suggesting that toddlers with higher PROCESS scores had larger discrepancies (i.e., different counts) of anticipatory saccades (i.e., those with more endorsed autism features showed greater change on this task than those with lower PROCESS scores, again possibly due to ceiling effects). There were no associations between the significant variables on the Cognitive Control tasks and average composite score, individual game scores, Mullen age equivalents, or ADOS‐2 severity scores (all *p*'s > 0.05).

###### 5.4.1.3.2 Joint Attention Task

On the Joint Attention task, change in responding on the book task was associated with total training time (rho = 0.50, *p* = 0.008), suggesting that toddlers who received more attention training were more responsive on the book task. There were no associations between the significant variables on the Joint Attention tasks and composite score, individual game scores, Mullen age‐equivalents, or ADOS‐2 severity scores (all *p*'s > 0.05).

##### 5.4.1.4 Assessment Scores

Relations between features of the training paradigm and clinical measures and each independent variable are included in Supporting Information S1.

## 6 Discussion

We examined differences across four attentional domains between toddlers with features of ASD who received an attention training intervention versus a control intervention, which yielded three main findings. First, as predicted, toddlers in the attention training group showed increased levels of responding to joint attention bids during an interactive task with an examiner compared to the control group and responded at higher rates of correct anticipatory saccades during a computer‐based cognitive control task. Second, participants in the attention training group did not differ from the control group on sustained attention or disengagement task performance. Third, change (i.e., improvements) in attention performance was associated with training dose, including the number of sessions attended and total training time, as well as higher Mullen Visual Reception subscale scores. Taken together, these results suggest that a relatively short attention training intervention influenced performance on some interactive and computer‐based measures of attention. Such attentional changes may have longstanding impacts on a range of domains, including language acquisition (Rose et al. 2009), academic learning (Scerif 2010; Welsh et al. 2010), and initiating and maintaining social interactions (Mundy et al. 2009).

All participants tolerated the *Attention Assessment* and intervention well. Previous research that has targeted attention has employed various parent‐ and teacher‐mediated behavioral interventions, yet intervention models specific to toddlers and very young preschoolers are limited and often focus on parenting skills (Ogundele and Ayyash 2023; Sibley et al. 2023). As such, we employed an attention intervention using computer‐based tasks based on looking; the child interacts with computer‐delivered stimuli that respond and adapt depending upon the child's gaze to the screen (i.e., is gaze‐contingent; Wass et al. 2011). This method is feasible and has been shown to be efficacious in very young typically developing infants (10–14 months), who demonstrated improvements in “sustained,” “distracted,” and “executive” attention following five training sessions (Wass et al. 2011). The feasibility of this method for use with autistic toddlers was assessed by Sacrey et al. (2023), who found that autistic toddlers tolerated the pre‐test portion of the *Attention Assessment* well. Toddlers in the attention and control portions of this intervention RCT attended similar numbers of sessions and contributed similar amounts of data at the pre‐ and post‐test *Attention Assessments*.

As expected, toddlers in the attention training group in the current study showed increased rates of joint attention behaviors during an interactive task with an examiner and higher rates of correct anticipatory saccades during the computer‐based cognitive control task. Showing transfer of training beyond the computer tasks, children in the attention group displayed increased bids for joint attention, as demonstrated by increased pointing to distal objects and pointing in imitation to the examiner's points, as well as responsivity to the examiner's points during the book task. With respect to the cognitive control task, toddlers in the attention training condition made more correct anticipatory saccades, as well as a higher proportion of correct anticipatory saccades compared to the control group. The pattern of improvements following the intervention was consistent with the findings of Wass et al. (2011), who reported improvements in correct anticipatory looks on the cognitive control task for neurotypical infants who received attention training. Similarly, our findings align with those of Forssman and Wass (2018), who found an increase in the proportion of responses to pointing on an interactive joint attention task but no differences on measures of eye contact. Attentional differences are a common feature of the autistic phenotype (Hours et al. 2022; Sacrey et al. 2014) and the results here suggest that joint attention and anticipatory saccades may be affected in ASD and are amenable to intervention.

Performance did not differ by condition on the sustained attention task, in which the child looks at static images of boring and interesting stimuli, nor on the task in which a child must visually disengage from a central stimulus to look at a peripheral stimulus. This lack of group differences runs contrary to our hypotheses. Previous research using this intervention with neurotypical infants (aged 10–14 months) reported improvements on measures of visual disengagement and sustained attention for infants who received the intervention versus a control condition (Wass et al. 2011). As such, we hypothesized that the intervention would improve performance on these tasks for toddlers with suspected or diagnosed autism. Our participants' age range may have contributed to this difference. Wass et al.'s participants were considerably younger and as such, may have had more capacity to develop following a brief intervention. Examination of the results supports this, as both groups in our study had shorter mean durations on baseline, gap, and overlap trials at pre‐intervention compared to post‐training intervention scores in Wass et al. (2011). Similarly, we saw lower peak durations on the sustained attention task (22 s for boring and 40 s for interesting trials) compared even to the post‐training intervention scores of participants in Wass et al. (25 s for boring and 47 s for interesting trials).

The lack of differences on the disengagement task and sustained attention tasks is in line with recent literature that suggest disengagement is differentially impacted by age. Sticky fixation, or difficulty disengaging from a visual stimulus, is commonly observed in autistic children and may vary with age (Sacrey et al. 2023). This has been seen on tasks of attentional disengagement, where they take longer to look away from a central stimulus on overlap trials compared to same‐aged neurotypical peers (Bryson et al. 2014; Elison et al. 2013; Elsabbagh et al. 2013; Zwaigenbaum et al. 2005). Other research has found no differences on the disengagement task between autistic and neurotypical participants (Fischer et al. 2014, 2016; Kikuchi et al. 2011; Wilson and Saldaña 2019). A key difference appears to be the age of the participants; children under 2 years of age have shown a disengagement delay, whereas studies with children at least 2 years old have shown no difference between autistic and neurotypical peers. Thus, “sticky fixation” in the disengagement task may be a transient phenomenon that improves during the first few years. Reviews by Colombo and Cheatham (2006) and Sacrey et al. (2014) support this proposition and note that disengagement takes a developmental U‐shaped course during the first 3 years of life. Factors that influence the developmental course of “sticky fixation” on the disengagement task may also influence looking patterns during the sustained attention task.

The strengths of the current study include the randomized controlled trial design and blind assessment of the effects of a brief, computer‐based intervention on multiple measures of attention in toddlers with early features of ASD. There are several limitations, however. First, our sample was relatively small, limiting the power to detect small to moderate differences. Data collection for this study was interrupted by the COVID‐19 pandemic; data collection and plans for long‐term follow‐up were interrupted. It would be important in the future to include a larger sample to allow a fulsome exploration of the effects of this intervention on attentional processes. Furthermore, it may be ideal to include an arm that explores the long‐term impacts of the attention intervention, with (and possibly without) an accompanying behavioral intervention (as was planned here). This would allow for the exploration of the development of toddlers with suspected or confirmed ASD to elucidate the differing impacts of attentional and behavioral interventions and combinations thereof. The attention training occurred over a brief period (6 weeks) and we did not control for potential maturation or practice effects. Nevertheless, both groups completed the pre‐ and post‐attention assessment, with the main difference of group participation being the responsive stimuli of the attention training games, which may lessen potential impacts of maturation/practice effects on the results. Second, our data analyses plan was not preregistered. Third, these findings may not generalize to children with ASD who may not be diagnosed until later in childhood or adulthood. Fourth, the attention intervention was administered to examine potential priming of the effects of a subsequent behavioral intervention (results to be presented in an upcoming manuscript). We used mixed models instead of *t*‐tests for the purpose of including pre‐test measures and significant demographic and clinical features as covariates, and as such, did not correct *p*‐values for the multiple comparisons, which may have resulted in Type I errors. This potential limitation is lessened due to the predicted impacts of attention training (e.g., differences on the computer‐based tasks and interactive tasks) based on previous literature prior to commencing the study. Fifth, our intervention group did have a higher percentage of participants with an older sibling with ASD, in which case they may present for clinical attention with somewhat milder symptoms. As such, the group differences in the post assessment may reflect this difference rather than an effect of treatment. Comparison of pre‐Attention Assessment results found no differences between toddlers with an older sibling with autism compared to those without, the smallest *p*‐value = 0.18. On clinical characteristics, toddlers with older siblings with autism were not different on the Mullen subscales, nor the PROCESS questionnaire; they did differ on the ADOS total severity score (*p* = 0.03) and SA severity score (*p* = 0.01), with the toddlers with older siblings with ASD having lower scores on both. We aimed to mitigate this difference by including pre‐assessment measures as covariates in the analyses. These results support the proposition that autistic individuals display an uneven cognitive profile (Bury et al. 2020) and children with an uneven profile may be identified earlier, providing an opportunity for this type of intervention. In the future, with a larger sample, it may be informative to incorporate more complex statistical analyses to identify potential subgroups of toddlers with different profiles. ASD is a heterogenous condition, and it is unlikely that the current (or indeed any) intervention protocol would benefit all toddlers equally. With such analyses, one may uncover a subgroup of toddlers who are more likely to benefit from the current intervention protocol (such as those with higher baseline expressive language) or who may benefit from more intensive intervention (e.g., lower baseline attention abilities).

Autism is associated with a cognitive profile characterized by relative strengths and challenges (Bury et al. 2020). As shown here, a brief, gaze‐contingent attention intervention can influence attentional performance. This finding is potentially clinically significant because skills gained during this computer‐based intervention were also evident in toddlers' performance during a face‐to‐face joint attention task with an examiner. It is important to note that this technological intervention method transferred to attentional performance in an interpersonal social interaction following only 4 weeks of training, independent of baseline performance. This approach has the potential to offer benefits for young children's developing attention skills, warranting further evaluation.

## Conflicts of Interest

The authors declare no conflicts of interest.

## Supporting information


**Data S1:** cdev70033‐sup‐0001‐supinfo.docx.

## Data Availability

Data are available by reasonable request to the first author.
