# Supplementary material for: Influence of a Short‐Term Attention Intervention on the Attentional Skills of Toddlers With Suspected or Confirmed Autism Spectrum Disorder
Source: Child Dev. 2025 Aug 22;96(6):2176–88. doi: 10.1111/cdev.70033 (PMC12598445; doi:10.1111/cdev.70033)
Supplement: Supplementary file 1 — Data S1: cdev70033‐sup‐0001‐supinfo.docx. [file CDEV-96-2176-s001.docx]

### Supplemental Materials

**Attention Intervention Tasks**

**Goal Maintenance**

***Indicators of performance***. We calculated the proportion of toddlers’ fixation to the target weighted by the difficulty level. Since more difficult levels presented more distracters, toddlers’ fixation to the target was given more weight when displayed while presented with more challenges.

***Task 1- Fly Me*:** A pink shape appeared on the screen: when the child fixated it, face-like features appeared on the shape (eyes and mouth) and it moved diagonally across the screen. Distractors appeared moving in different directions. When the child looked away from the target character, it stopped moving and its face-like features faded out while at the same time the distractors faded out from the screen. The target character resumed moving and its face-like features reappeared when the child fixated it again. The distractors changed adaptively.

***Task 2 - Butterfly****:* A butterfly was presented on the screen. When the child fixated the butterfly, the butterfly flied across the screen, while distractors (a house, a tree, clouds) scrolled in the opposite direction. When the child looked to any of the distractors, they disappeared and only the butterfly, now static, remained on screen. On re-fixating the butterfly, it re-commenced moving and the distractors re-appeared and continued scrolling. The salience of the distractors changed adaptively, including faster, larger and more densely packed objects.


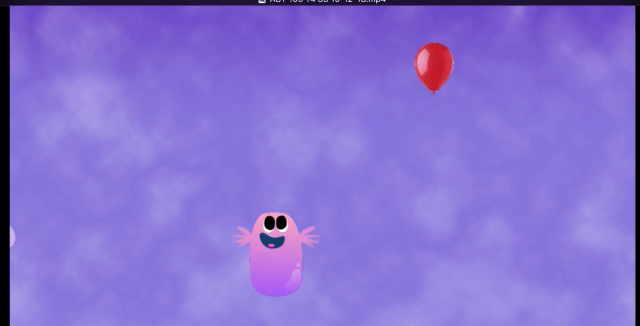


Fly Me Butterfly

**Working Memory**

***Performance indicator***: We calculated the reciprocal of the response time taken to look at the correct target. Thus, toddlers who were faster in meeting task demands obtained higher scores.

**Task 1- Puzzle Memory**: A character moved into one out of two (or more) squares, then disappeared behind a screen. The screen then became black, and a central stimulus appeared (a ball moving from top midline to bottom) in order to attract toddlers’ attention. After this, the two squares reappeared covered by the screen. After the toddler looked at the square where this character had been, the character was revealed and stayed inside the square. A new character appeared, moved into the other square, the screen became black, and the central stimulus (moving ball) was displayed. After this, the two squares reappeared: the first character was still visible inside the square, while the other square was covered by the screen. When the toddler looked at the square where the novel character was, the character popped out producing a sound.

**Task 2 - Windows**: When the toddler fixated the target (an animal in a window), an animation showed the target disappearing into one of several windows, which were then covered with curtains. A fixation target (a flower) appeared elsewhere on the screen and rotated when the toddler looked at it. After a delay period, the fixation target disappeared. If the toddler looked back to the window behind which the target had disappeared, they received an animation as a reward. The number of windows, the salience of the distractors, and the length of the delay changed adaptively.

**Task 3 – Tausendfuss:** An animated character appeared inside a square, moved and made a sound, then disappeared behind a screen that covered both squares. A central stimulus than appeared in the top midline of the screen (a moving spiral). If the toddler looked back at the square where the animated character had appeared, the character popped out and made a noise. Following this, when the toddler looked at the other empty square, a new animated character appeared. This was also covered by a screen, while the first character remained visible in the other square. A central stimulus appeared again. After this, the second novel character popped out of the covered square if the toddler looked at it for a pre-defined amount of time.

**Task 4 – 3 Little Maids:** A character appeared on the screen, which was successively covered by one of two (or more) pots that moved into the screen. After this, a central fixation target appeared on the screen to attract the toddlers’ attention to midline (a moving cartoon spider). After the central fixation stimulus disappeared, the child was rewarded by an animation if she looked back (within a pre-set time limit of 10 s) to the pot in the location where the original character had been. The task changed adaptively by increasing the number of pots on the screen from which to select the target.


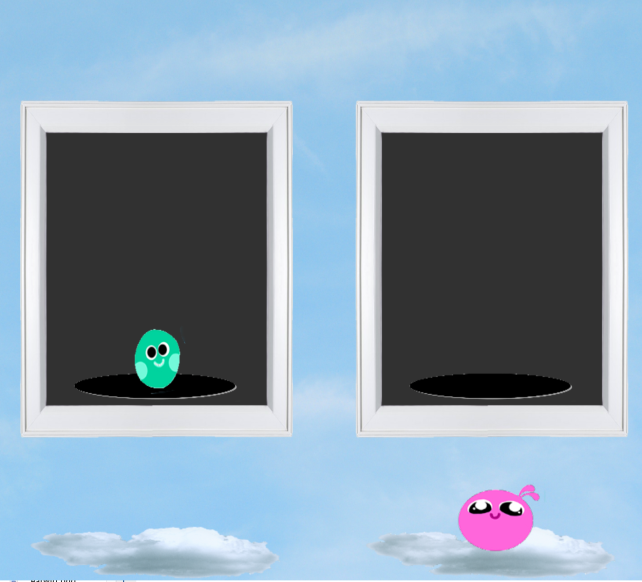

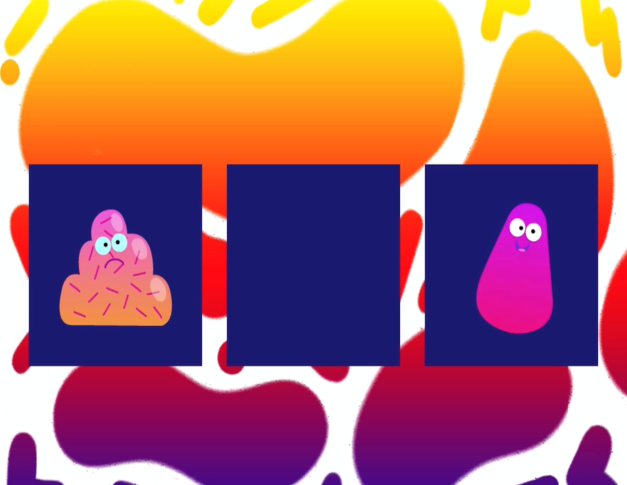

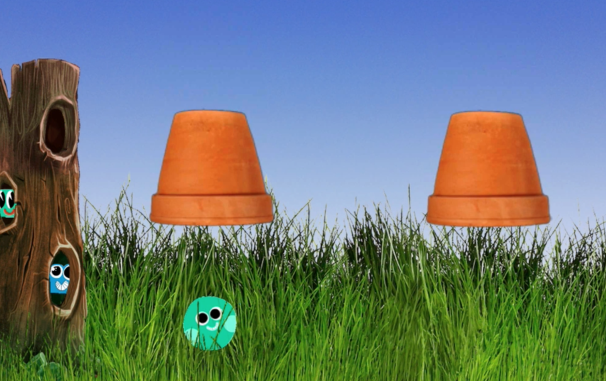


Puzzle Memory Windows Tausendfuss 3 Little Maids

**Target Searching**

***Performance indicator*:** We calculated the reciprocal of the response time toddlers employed in finding the correct target weighted by the difficulty level. In this way, toddlers obtained higher scores for succeeding in the task faster when the task presented higher demands.

***Task 1 – Stars****:* One of five possible targets (cartoon characters in brightly coloured stars) were presented on screen together with eight distractors (smaller stars, planets, clouds). If the toddler looked to the target within 3000 ms, she received an animation as a reward. The target changed from trial to trial. The salience of the distractors changed adaptively. At lower difficulty levels, the eight distractors were smaller, static, and identical to each other and dissimilar from the targets. At higher difficulty levels, they were more varied, moving, brightly coloured, and similar to the targets.

***Task 2 - Suspects****:* One of two possible targets (either an elephant or a chicken) was presented with one or more distractor items of the same size. When the toddler looked at the target within a time limit, she received an animation as a reward. The same target was then re-presented with other distractor(s). The number of distractors varied adaptively with performance; at higher performance levels, more distractors were presented. Between blocks of 12 trials, the target changed: where previously the toddler had received a reward for looking to the elephant, she was successively rewarded for looking to the chicken. At higher difficulty levels, the target from the previous block was presented concurrently with the target from the current block (a conflict trial); at lower difficulty levels, only novel distractors were presented (non-conflict).

***Task 3 – Disengagement:*** A central moving stimulus appeared on the screen (a colourful flower), and after that, two characters appeared. If the toddler watched at one of the two chosen at random by the computer script, a rewarding animation was displayed. In successive trials, the toddler received the animation every time she looked at the pre-set target character. The number of distractors increased adaptively.


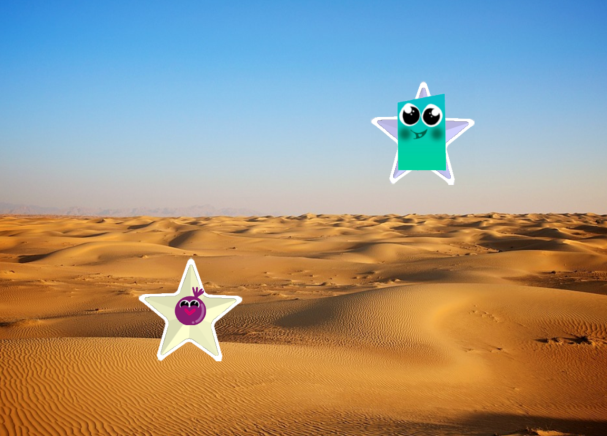


Stars Suspects Disengagement

***Pre-Attention Assessment***

Comparison on pre-*Attention Assessment* resulted in group differences on two of the four attention tasks. There were group differences on the Joint Attention task for pointing (*p* = .04), pointing with eye contact (*p* = .02), pointing in book (*p* = .008), and pointing in imitation (*p* = .009). On the Sustained Attention task, there were group differences on the range of looking during the interesting trials (*p* = .04). There were no group differences on the Cognitive Control or Disengagement Tasks. All pre-*Attention Assessment* variables were included as covariates in the post-*Attention Assessment* analyses regardless of if they were significantly different at baseline.

**Table S1: Results from Correlations between Joint Attention and Mullen Age Equivalents**

| Joint Attention Measure | Visual  Reception | Fine  Motor | Receptive Language | Expressive Language |
| --- | --- | --- | --- | --- |
| Eye contact | **r = .26, p = .05*** | **r = .35, p = .03 *** | **r = .52, p < .001 ***** | **r = .27, p = .04 *** |
| Alternate | **r = .30, p = .03*** | **r = .34, p = .04*** | **r = .34, p = .03*** | **r = .41, p = .002**** |
| Low IJA Total | **r = .37, p = .005**** | **r = .42, p = .01**** | **r = .50, p = .001***** | **r = .40, p = .002**** |
| Point | **r = .41, p = .002**** | **r = .36, p = .03*** | **r = .47, p = .003**** | **r = .52, p < .001***** |
| Point + eye contact | **r = .39, p = .003**** | **r = .44, p = .006**** | **r = .46, p = .003**** | r = .25, p = .07 |
| Show | r = .09, p = .52 | r = .23, p = .17 | r = .11, p = .51 | r = -.05, p = .69 |
| High IJA Total | **r = .49, p < .001***** | **r = .54, p < .001***** | **r = .56, p < .001***** | **r = .53, p < .001***** |
| Bid to caregiver | r = .20, p = .15 | r = .21, p = .20 | r = .23, p = .16 | **r = .39, p = .003**** |
| Book task | **r = .47, p < .001***** | **r = .60, p < .001***** | **r = .57, p < .001***** | **r = .38, p = .004**** |
| Near posters | **r = .35, p = .007**** | r = .25, p = .12 | **r = .42, p = .007**** | **r = .28, p = .03*** |
| Far posters | r = .21, p = .88 | r = -.09, p = .59 | r = -.09, p = .59 | r = .02, p = .87 |
| Point in imitation | **r = .55, p < .001***** | **r = .60, p < .001***** | **r = .53, p < .001***** | **r = .49, p < .001***** |

Significance: * < .05; ** < .01; Abbreviations: IJA = initiating joint attention

Abbreviation; IJA = Initiating Joint Attention

The data for each variable were visualized using box plot in Stata IC 15.1.


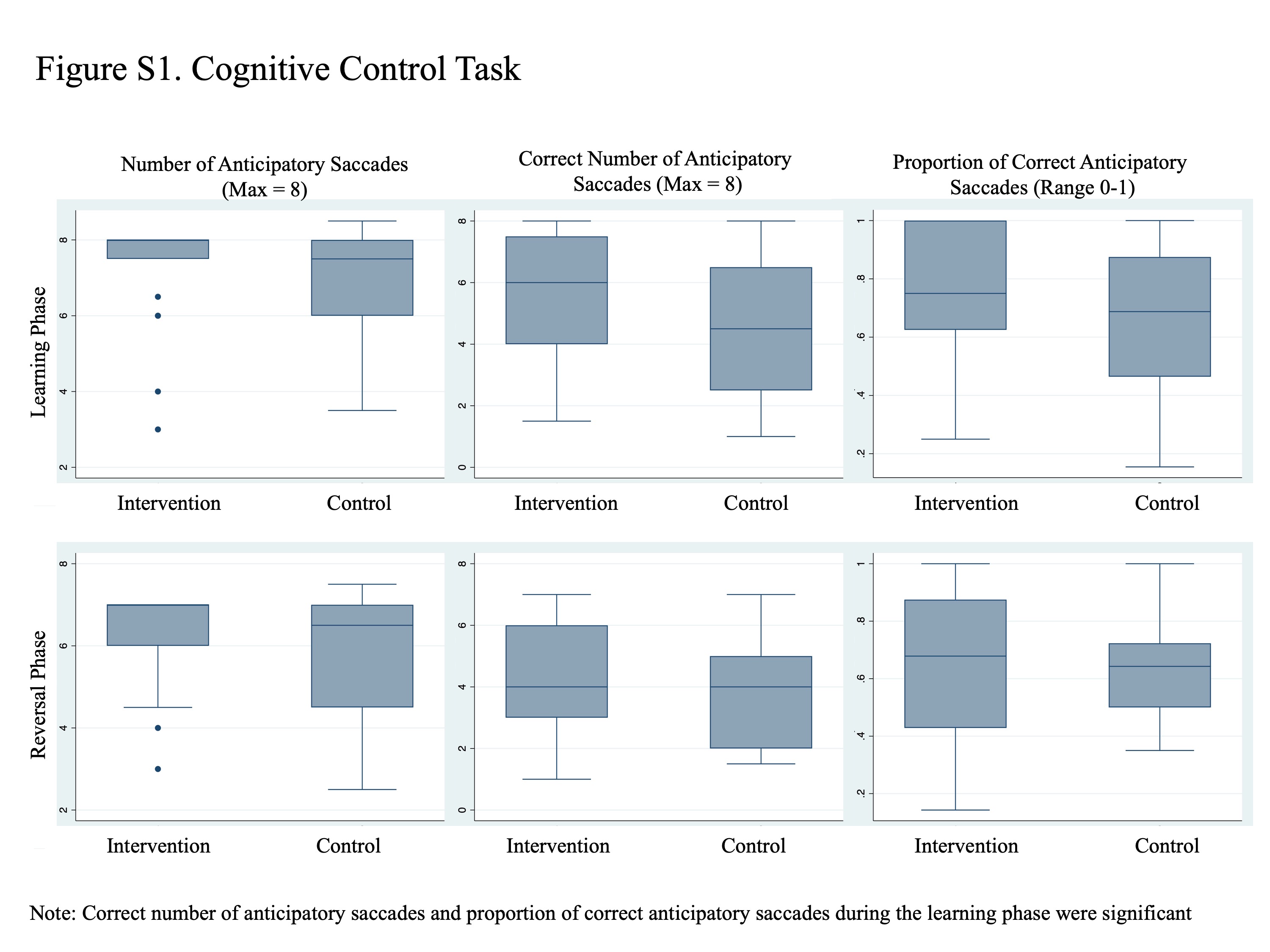


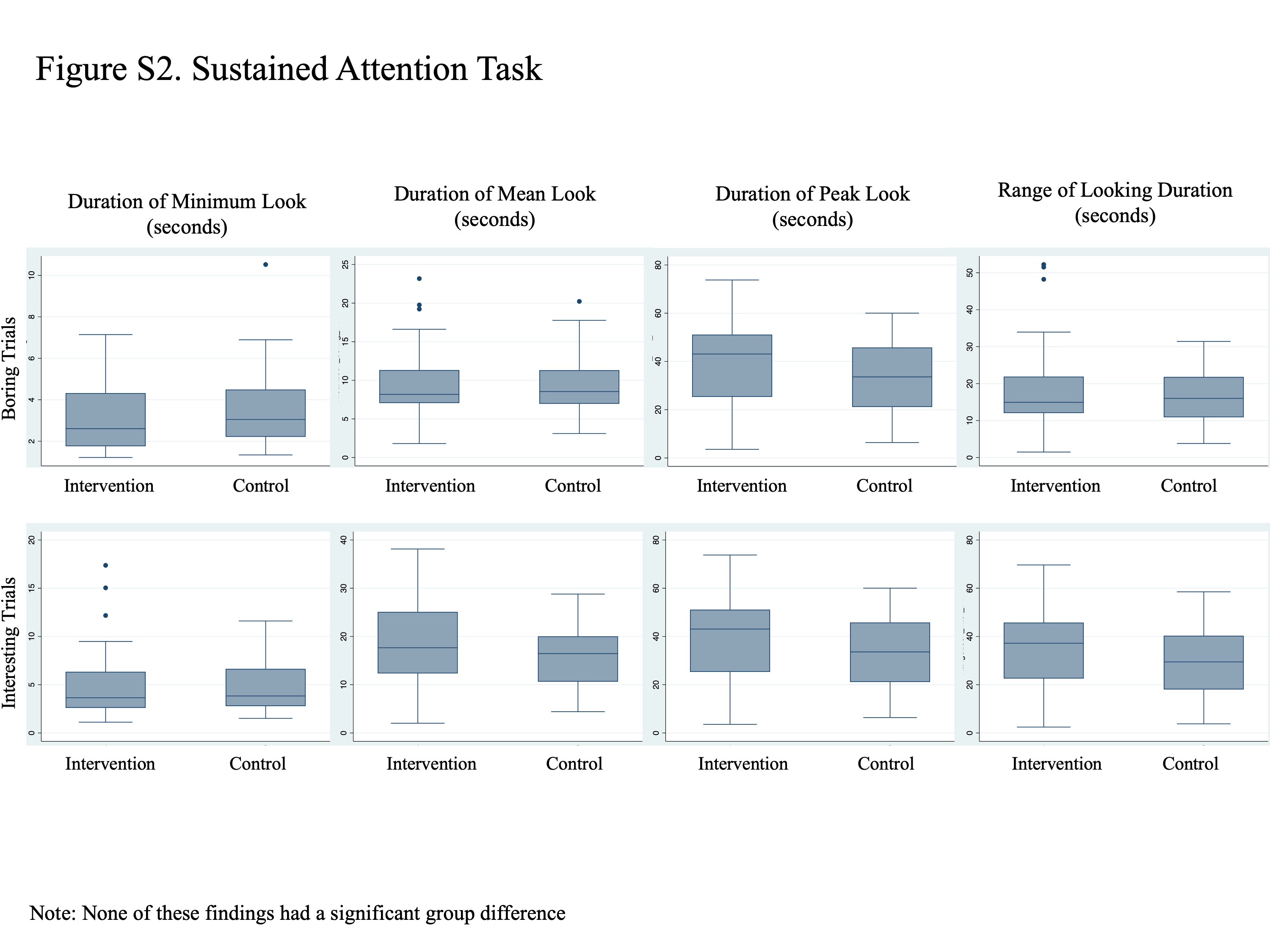

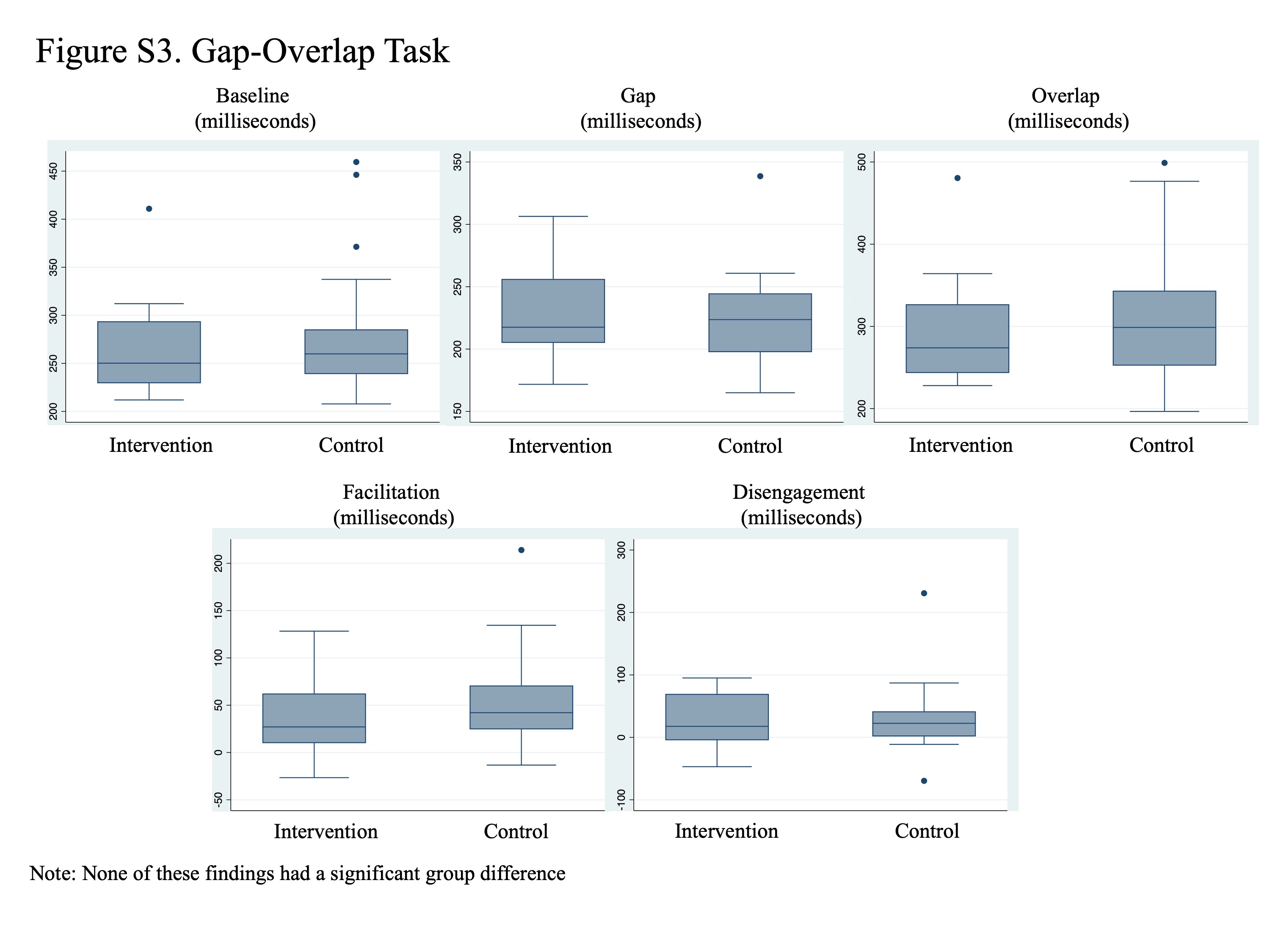

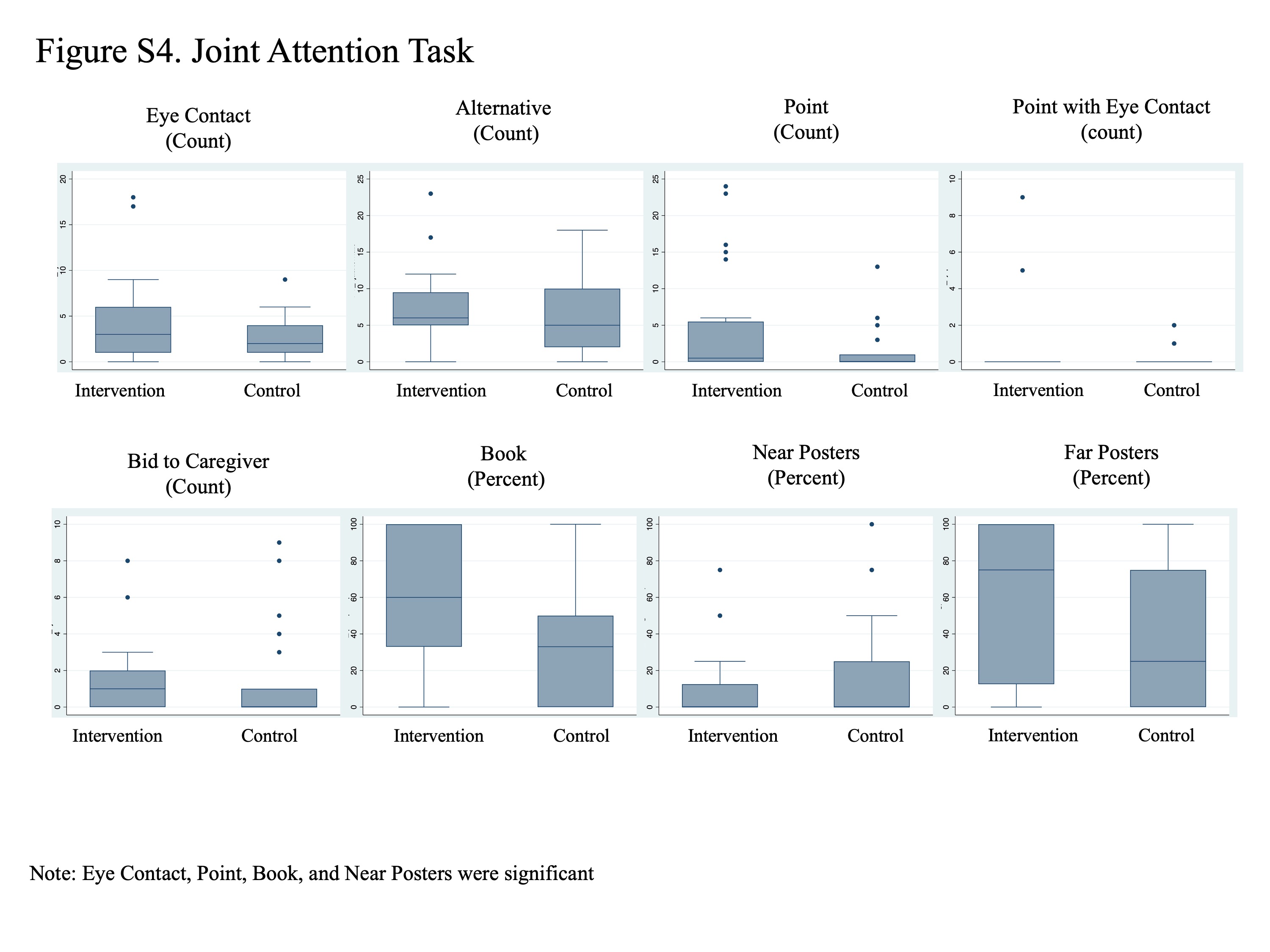


**Table S2: Results from Mixed Model Analyses**

| Post Measure | Group | Pre  Measure | Mother  Ethnicity |
| --- | --- | --- | --- |
| Joint Attention | | | |
| Eye contact | *F*(1,52) = 3.35, *p* = .07 | X | - |
| Alternate | *F*(1,52) = 1.15, *p* = .29 | X | X |
| Low IJA Total | *F*(1,52) = 3.07, *p* = .09 | X | - |
| Point | ***F*(1,52) = 5.11, *p* = .03*** | X | - |
| Point + eye contact | *F*(1,52) = .54, *p* = .47 | X | - |
| Show | *F*(1,52) = .42, *p* = .52 | X | - |
| High IJA Total | *F*(1,52) = .001, *p* = .98 | X | - |
| Bid to caregiver | *F*(1,52) = .08, *p* = .78 | X | - |
| Book task | ***F*(1,48) = 5.25, *p* = .03*** | X | X |
| Near posters | ***F*(1,54) = 4.17, *p* = .04*** | X | - |
| Far posters | *F*(1,54) = .16, *p* = .70 | X | - |
| Point in imitation | ***F*(1,53) = 4.22, *p* = .04*** | X | - |
| Disengagement Task | | | |
| Baseline SRT mean | *F*(1,31) = 1.32, *p* = .26 | X | - |
| Gap SRT mean | *F*(1,31)= .03, *p* = .86 | X | - |
| Overlap SRT mean | *F*(1,29) = .88, *p* = .36 | X | - |
| Facilitation SRT mean | *F*(1,31) = 1.83, *p* = .19 | X | - |
| Disengagement SRT mean | *F*(1,31) = .51, *p* = .48 | X | - |
| Sustained Attention | | | |
| Peak look - boring | *F*(1,63) =.46, *p* = .50 | X | - |
| Mean look - boring | *F*(1,63) =.25, *p* = .62 | X | - |
| Minimum look - boring | *F*(1,63) =.75, *p* = .39 | X | - |
| Range look - boring | *F*(1,63) =.75, *p* = .39 | X | - |
| Peak look - interesting | *F*(1,60) = 2.85, *p* = .10 | X | - |
| Mean look - interesting | *F*(1,60) = 3.12, *p* = .08 | X | - |
| Minimum look - interesting | *F*(1,60) =.44, *p* = .51 | X | - |
| Range look - interesting | *F*(1,60) = 2.68, *p* = .11 | X | - |
| Cognitive Control | | | |
| Number anticipatory saccade - learn | *F*(1,56) = 2.08, *p* = .16 | X | - |
| Number correct anticipatory saccade - learn | ***F*(1,56) = 5.30, *p* = .03*** | X | - |
| Proportion correct anticipatory saccade - learn | ***F*(1,56) = 4.58, *p* = .04*** | X | - |
| Number anticipatory saccade - reversal | *F*(1,54) = 3.83, *p* = .06 | X | - |
| Number correct anticipatory saccade - reversal | *F*(1,54) =1.09, *p* = .30 | X | - |
| Proportion correct anticipatory saccade - reversal | *F*(1,54) =.04, *p* = .85 | X | - |

Abbreviations: IJA = initiating joint attention; SRT = saccadic reaction time

Significance: * < .05; ** < .01; X = included as covariate

**Contributors of Attention Training Performance**

For the attention training group, parameters of the intervention and clinical characteristics of the participants were explored to determine potential drivers of changes. Change scores on the *Attention Assessment* intervention (i.e., post score – pre score = change score) were correlated with features of the training environment, including total training time, average training per game, number of games completed, and number of sessions completed. Additionally, clinical characteristics, including Mullen age equivalents, ADOS total severity score, and total score on PROCESS were correlated with change scores on the *Attention Assessment.*

With respect to the intervention, **average change in performance across training** was associated with pointing with eye contact (*rho* = -.41, *p* = .02) and pointing in imitation (*rho* = -.48, *p* = .006) on the Joint Attention task, baseline SRT (*rho* = .59, *p* = .01) and overlap SRT (*rho* = .46, *p* = .05) on the Disengagement task, and number of correct anticipatory saccades during reversal (*rho* = .45, *p* = .01) and proportion of correct anticipatory saccades during reversal (*rho* = .54, *p* = .002) on the Cognitive Control task. **Total training time** was associated with showing objects (*rho* = -.47, *p* = .008), bids to caregiver (*rho* = .37, *p* = .03), response during the book task (*rho* = .37, *p* = .03) on the Joint Attention task, baseline SRT (*rho* = -.57, *p* = .01) on the Disengagement task, peak look during boring trials (*rho* = .43, *p* = .007) and range of looking during boring trials (*rho* = .47, *p* = .003) on the Sustained Attention task, and proportion of correct anticipatory saccades during the reversal phase (*rho* = .33, *p* = .04) on the Cognitive Control task. **Average training per game** was associated with gap SRT (*rho* = -.48, *p* = .03) on the Disengagement task, as well as reaction time during the learning phase (*rho* = -.33, *p* = .05) on the Cognitive Control task. **Total number of games played** was associated with pointing with eye contact (*rho* = -.35, *p* = .04), showing objects (*rho* = -.47, *p* = .007), responding to points in the book task (*rho* = .51, *p* = .004), and responding to far posters (*rho* = .46, *p* = .03) on the Joint Attention task, peak look during boring trials (*rho* = .40, *p* = .01) and range of looking during boring trials (*rho* = .41, *p* = .01) on the Sustained Attention task, as well as number of correct anticipatory saccades during learning (*rho* = -.38, *p* = .03) on the Cognitive Control task. **Number of sessions completed** was associated with showing objects (*rho* = -.44, *p* = .01) and responding to points in the book task (*rho* = .37, *p* = .03) on the Joint Attention task, baseline SRT (*rho* = -.48, *p* = .03) and gap SRT (*rho* = -.64, *p* = .004) on the Disengagement task, as well as minimum look during boring trials (*rho* = -.37, *p* = .02) on the Sustained Attention task.

With respect to Mullen age equivalents (AE), **visual reception AE** was associated with pointing in imitation (*rho* = -.44, *p* = .01) on the Joint Attention task, as well as peak looking during interesting trials (*rho* = .56, *p* < .001), mean look during interesting trials (*rho* = .50, *p* = .003), and range of looking during interesting trials (*rho* = .53, *p* = .002) on the Sustained Attention task. **Fine motor AE** was associated with pointing in imitation (*rho* = -.45, *p* = .02) on the Joint Attention task, peak looking during interesting trials (*rho* = .54, *p* = .007), mean look during interesting trials (*rho* = .52, *p* = .01), and range of looking during interesting trials (*rho* = .55, *p* = .006) on the Sustained Attention task, as well as number of anticipatory saccades during learning (*rho* = -.44, *p* = .03), and mean reaction time for correct anticipatory saccades during learning (*rho* = .50, *p* = .01) and reversal (*rho* = -.45, *p* = .03) phases of the Cognitive Control task. **Receptive Language AE** was associated with pointing (*rho* = .45, *p* = .02) and pointing in imitation (*rho* = -.53, *p* = .009) on the Joint Attention task, as well as peak looking during interesting trials (*rho* = .74, *p* < .001), mean look during interesting trials (*rho* = .59, *p* = .004), and range of looking during interesting trials (*rho* = .73, *p* < .001) on the Sustained Attention task. **Expressive Language AE** was associated with peak looking during interesting trials (*rho* = .45 *p* = .009), mean look during interesting trials (*rho* = .37, *p* = .03), and range of looking during interesting trials (*rho* = .39, *p* = .02) on the Sustained Attention task.

With respect to the features of autism, the **ADOS total severity** score was associated with alternating eye contact (*rho* = -.47, *p* = .008) on the Joint Attention task, peak look during interesting trial (*rho* = -.58, *p* < .001), mean look during interesting trials (*rho* = -.59, *p* < .001), and range of looking during interesting trials (*rho* = -.53, *p* = .002) on the Sustained Attention task, as well as number of correct anticipatory saccades during the reversal phase (*rho* = -.34, *p* = .04) and mean reaction time for correct anticipatory saccades during the reversal phase (*rho* = .36, *p* = .04) of the Cognitive Control task. The **PROCESS total score** was associated with gap SRT (*rho* = -.54, *p* = .02) and overlap SRT (*rho* = -.51, *p* = .03) on the Disengagement task, peak look during boring trials (*rho* = .34, *p* = .03) and range of looking during boring trials (*rho* = -.38, *p* = .02) on Sustained Attention task, as well as number of correct anticipatory saccades during learning (*rho* = .41, *p* = .02) and reversal (*rho* = -.37, *p* = .03) phases and mean reaction time for correct anticipatory saccades during the reversal phase (*rho* = .44, *p* = .02) of the Cognitive Control task.
